# Ectopic bronchogenic cyst in the retroperitoneal region: a case report and literature review of adult patients

**DOI:** 10.1186/s12893-021-01341-w

**Published:** 2021-09-20

**Authors:** Kaitao Yuan, Man Shu, Yan Ma, Weidong Feng, Jinning Ye, Yujie Yuan

**Affiliations:** 1 Center of Gastrointestinal Surgery, The First Affiliated Hospital, Sun Yat-sen University, No. 58 2nd Zhongshan Road, Guangzhou, 510080 Guangdong Province People’s Republic of China; 2 Department of Pathology, The First Affiliated Hospital, Sun Yat-sen University, Guangzhou, 510080 Guangdong Province People’s Republic of China

**Keywords:** Retroperitoneal neoplasm, Bronchogenic cyst, Case report, Literature review, Surgical management

## Abstract

**Background:**

Bronchogenic cyst is congenital aberration of bronchopulmonary malformation, which is rarely encountered in the abdomen and retroperitoneum. We present a case report and literature review of retroperitoneal bronchogenic cyst.

**Case presentation:**

A 53-year-old female presented to outpatient clinic for a routine checkup of lumbar intervertebral disc herniation. She received a contrast computed tomography scan of the abdomen which revealed a retroperitoneal cystic lesion below the left crura of diaphragm. Afterward, the patient underwent a laparoscopic excision of the cystic lesion and was discharged uneventfully at postoperative day 4. Histopathological findings confirmed the diagnosis of retroperitoneal bronchogenic cyst. Our literature review identified 55 adult cases in recent two decades. The average age at diagnosis was 43.2 (range 17–69) years. 44 (80%) cases had a retroperitoneal cyst on the left side, and 52 (94.5%) cases underwent curative excision through open or laparoscopic surgery. In the available follow up of cases, there was no recurrence after surgery.

**Conclusions:**

Bronchogenic cyst is rare in the retroperitoneal region. It should be considered as one of the differential diagnoses of a retroperitoneal neoplasm.

## Background

Bronchogenic cyst is rare, benign congenital malformation of the tracheobronchial tree within the early embryologic foregut period [1]. It is mostly discovered in the posterior mediastinum but rarely found in the retroperitoneal region. Retroperitoneal bronchogenic cyst is first reported by Miller et al. in 1953 [2]. Since such cyst is usually asymptomatic, its diagnosis before surgery in such location is still challenging. We here present a woman with incidental detection of a retroperitoneal bronchogenic cyst, which was successfully managed through a laparoscopic excision. Additionally, we have performed a literature review to update the clinical features of this rare disease in adult patients.

## Case presentation

**Fig. 1:**
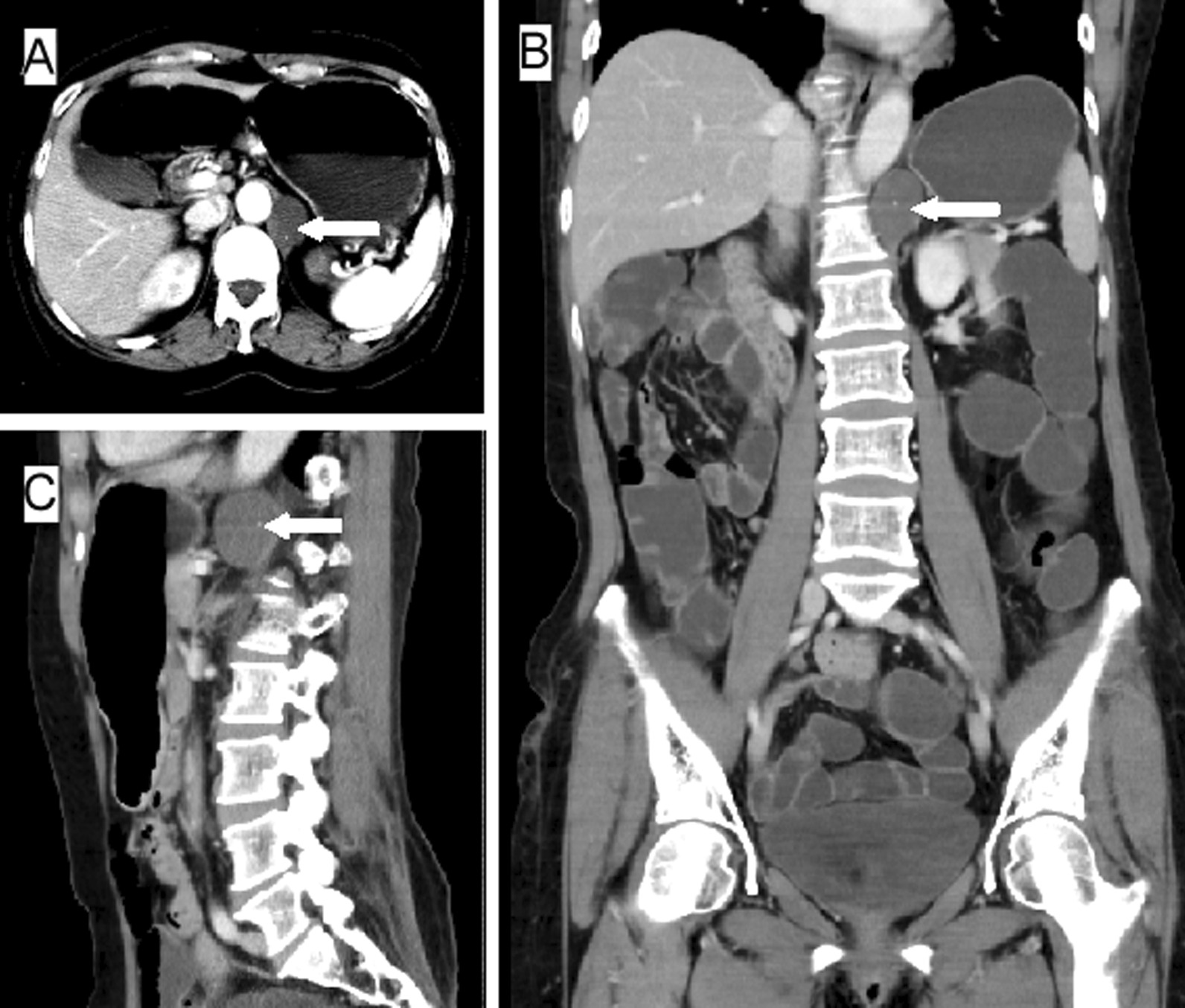
Radiological findings of the reported case. Contrast-enhanced CT scan shows a thin-walled water-attenuated cystic lesion in retroperitoneal region (white arrows). Panel **A**, Axial view; Panel **B**, Coronal view; Panel **C**, Sagittal view

**Fig. 2:**
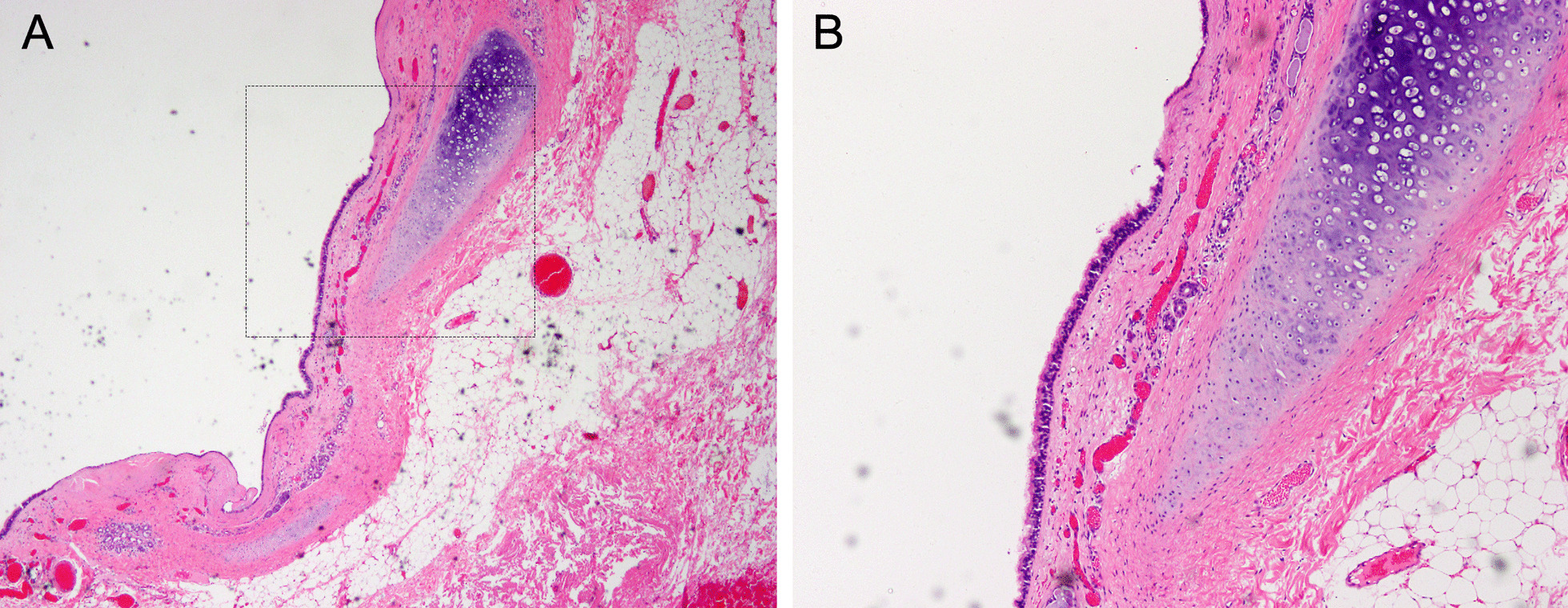
Microscopic appearance of retroperitoneal bronchogenic cyst. The cystic wall was consisted of pseudostratified epithelium, smooth muscle, mixed seromucous glands and cartilage. (Hematoxylin and eosin stain, original magnification: **A** ×40 and **B** ×200 power fields; The dotted box indicates magnified field)

A 53-year-old female patient presented to the outpatient clinic of local hospital in June 2017 (day 0) for routine checkup of lumbar intervertebral disc herniation. She underwent a magnetic resonance imaging (MRI) scan that revealed a round small mass in the retroperitoneal space. On day 3, she was referral to our department for further diagnosis and treatment of the retroperitoneal neoplasm. She did not have any complaint except for disc herniation-related low back pain. Her past history and family history were non-contributory. Physical examination was insignificant. Routine laboratory studies were normal. Specifically, serum tumor markers including carbohydrate antigen 19-9 (CA19-9), carbohydrate antigen 125 (CA125) and carcinoembryonic antigen (CEA) were all within normal ranges. Afterward, she received abdominal contrast-enhanced computed tomography (CT) scans, which revealed a well circumscribed cystic lesion, measuring 3.3 × 2.7 × 3.5 cm^3^ and filling with non-enhancing fluid-density collections, in her retroperitoneal region (Fig. 1). The lesion was located below the diaphragm and was adjacent to the left crura of diaphragm and abdominal aorta. After a multidisciplinary team discussion, a minimally invasive surgery was planned for her to determine the feature of such cystic lesion. On day 12, the patient underwent laparoscopic exploration and lesion excision. The cystic lesion, smoothly surfaced with mucinous content, was totally separated from the left crura of diaphragm, abdominal aorta, stomach, and left adrenal gland. The operation was successfully performed, which lasted for 90 min with estimated blood loss 10 mL. Oral feeding started 6 h after surgery, and the postoperative recovery was uneventful. On day 16, the patient was discharged uneventfully from the hospital. She did not develop any complications at 3-month follow-up. Her histopathological findings indicated that the cystic wall consisted of ciliated pseudostratified epithelium, smooth muscle, seromucous glands and fully developed cartilage (Fig. 2). Thus, the patient had a confirmed diagnosis of retroperitoneal bronchogenic cyst. She continued her daily activities without any limitation and had no evidence of recurrence within two years of follow-up.

## Discussion and conclusions

Bronchogenic cyst was defined with the following criteria: pseudo-stratified, ciliated, columnar epithelium together with the existence of at least one of the following structures: cartilage, smooth muscle or seromucous glands [3]. It commonly occurred in the mediastinal region of the thorax, while rarely located in the retroperitoneal region. To review this uncommon disease, we searched PubMed and Embase databases for similar case reports published between January 1998 and January 2018. The used keywords were as follows: “retroperitoneal”, “bronchogenic”, “mass”, “tumor”, “neoplasm” and “cyst”. English language studies with adult patient populations (≥ 18 years) were reviewed by two surgeons (WF and JY). The clinical data of selected cases with special attention to primary symptoms, tumor size and location, and histopathological features were summarized.

In sum, 55 cases (24 female and 31 male) with retroperitoneal bronchogenic cyst were identified within the study period. The clinical characteristics of included cases are shown in Table 1. The average age at diagnosis of retroperitoneal bronchogenic cyst was 43.2 (range 17–69) years old. The primary complaints included asymptomatic (27 cases), abdominal discomfort (12 cases), left flank pain (7 cases) and back pain (5 cases). The average diameter of retroperitoneal bronchogenic cyst was 6.4 (range 2–20) cm. Most of those cysts (44 cases) were found in the left retroperitoneal region, with eight cases discovered on the right side. The serum level of CA19-9 was elevated in only two cases [4, 5]. There was one case reported CEA level elevated inside the cyst but normal in the serum [6]. Laparoscopic resection was performed in most of the reported patients as the increased popularity of minimally invasive surgery. There were only two cases who received biopsy procedures and routine follow-up visits [7, 8]. For those obtaining a curative resection, postoperative recurrence was not reported.

**Table 1:** Literature review of retroperitoneal bronchogenic cyst

No.	Study	Country	Gender	Age	Primary symptoms	Maximal size (cm)	Location	Treatment	Specific features
1	Buckley 1998 [16]	USA	F	46	Epigastric pain and weight loss	4	Left adrenal gland	Laparotomy	
2	Yamamoto 1998 [17]	Japan	F	49	No	3	Right adrenal glad	Laparoscopic	
3	Itoh 1999 [18]	Japan	F	46	Pain in the left flank	8	Left adrenal glad	Laparotomy	
4	Sullivan 1999 [19]	Japan	F	55	Lower abdominal discomfort	10	Retroperitoneal to ascending colon	Laparotomy	
5	Yang 1999 [20]	South Korea	M	30	No	6	Left adrenal glad	Laparotomy	
6	Reichelt 2000 [21]	Germany	M	46	No	3.8	Right retroperitoneum	Laparotomy	
7	Haddadin 2001 [22]	UK	M	51	Epigastric pain	4	Left suprarenal region	Laparotomy	
8	Anderson 2001 [23]	USA	M	33	Left flank pain and gross hematuria	6	Left suprarenal region	Laparotomy	Adenocarcinoma with P53 positive
9	Martin 2002 [24]	Spain	M	51	No	8	Left diaphragmic pillar	Laparotomy	
10	Ingu 2002 [25]	Japan	F	46	Progressive left-arm numbness	4	Left hemidiaphragm	Laparotomy	
11	Andersson 2003 [26]	Sweden	M	38	Upper abdominal pain and weight loss	4.5	Left posteriors to pancreas	Laparotomy	No recurrence 2 years follow-up
12	Ishikawa 2003 [15]	Japan	F	41	Left flank pain	9.2	Left adrenal gland	Retroperitoneoscopic	
13	Hedayati 2003 [27]	USA	F	59	No	7	Left adrenal gland	Laparotomy	
14	Hisatomi 2003 [28]	Japan	M	42	Left flank pain	12	Left retroperitoneal space	Laparotomy	
15	Ishizuka 2004 [29]	Japan	M	36	No	5	Left adrenal glad	Laparoscopic	
16	Goh 2004 [30]	Singapore	F	29	A right-sided abdominal mass	18.9	Right retroperitoneal region	Laparotomy	
17	Paik 2005 [31]	South Korea	M	59	No	7	Superior border of pancreas neck	Laparotomy	Colon cancer
18	Wang 2006 [32]	Taiwan	M	69	Right upper quadrant discomfort	7	Left anterior pararenal space	Laparotomy	
19	Kim 2007 [33]	South Korea	M	17	Abdominal pain	3.2	Left anterior pararenal space	Laparotomy	
20	Chu 2007 [34]	Taiwan	M	55	No	4	Left adrenal gland	Laparoscopic	
21	Roma 2008 [35]	USA	M	40	No	6.2	Left adrenal gland	Laparoscopic	
22	Chung 2009 [36]	South Korea	F	41	No	4.8	Left adrenal gland	Retroperitoneoscopic	
23	Obando 2009 [37]	USA	M	67	No	3.9	Between pancreas and stomach	Laparoscopic	
24	Önol 2009 [38]	Turkey	M	36	No	6	Left adrenal	Laparotomy	
25	Inaba 2010 [39]	Japan	F	64	No	4	Posterior wall of stomach	Laparoscopic	
26	El Youssef 2010 [40]	Portland	M	44	No	3	Left adrenal mass	Laparoscopic	
27	Diaz 2010 [41]	Spain	M	67	Low back pain	6	GEJ to left diaphragmatic crura	Laparoscopic	
28	Petrina 2010 [42]	Italy	M	33	Epigastric abdominal pain	5	Ileal mesentery	Laparotomy	
29	Alguraan 2012 [43]	USA	F	23	No	4	Right adrenal mass	Retroperitoneoscopic	Robotic excision
30	Parray 2012 [44]	India	F	30	Right upper quadrant pain	10	Right suprarenal area	Laparotomy	
31	O'Neal 2012 [45]	USA	F	23	Vague abdominal discomfort	5.2	Left adrenal gland	Retroperitoneoscopic	
32	Brient 2012 [7]	France	M	60	No	NA	Left diaphragmic pillar	CT-guided biopsy	No recurrence 3 years follow-up
33	Govaerts 2012 [2]	Belgium	M	48	No	7.5	Left adrenal glad	Laparotomy	
34	Choi 2012 [14]	South Korea	F	45	Intermittent abdominal discomfort	8	Left adrenal gland	Laparotomy	
35	Runge 2013 [6]	Switzerland	F	42	Epigastric pain	5.0	Left adrenal glad	Laparoscopic	Inside CEA 3777 μg/L
36	Cai 2013 [46]	China	F	26	No	4	Left adrenal region	Laparotomy	
37	Castro 2013 [47]	Portugal	F	36	Abdominal pain	8	Left upper retroperitoneum	Laparoscopic	
38	Jannasch 2013 [4]	Germany	M	50	In the left flank pain	4	Left adrenal region	Retroperitoneoscopic	CA19-9 144.1
39	Kluger 2013 [48]	USA	F	49	Intermittent epigastric pain	8.5	Right side of pancreas head	Laparotomy	Whipple’s procedure
40	Cao 2014 [9]	China	M	51	Intermittent fatigue	2.1	Bilateral adrenal	Retroperitoneoscopic	
41	Dong 2014 [49]	China	F	30	No	2	Left suprarenal region	Retroperitoneoscopic	CEA elevated inside
42	Mirsadeghi 2014 [10]	Iran	M	23	Left upper quadrant pain	20	Between spleen and left kidney	Laparotomy	No recurrence 4 years follow-up
43	Terasaka 2014 [50]	Japan	M	27	No	5.4	Left adrenal gland	Laparoscopic	
44	Bulut 2015 [51]	Turkey	F	25	Pain in the left flank	4	Left adrenal gland	Laparoscopic	
45	Herek 2015 [52]	Turkey	M	42	Back pain	9	Left diaphragmic crura	Laparotomy	
46	Jiang 2015 [53]	China	M	52	No	2.5	Left retroperitoneal space	Retroperitoneoscopic	
47	Robertson 2015 [54]	UK	M	56	Right hypochondrial pain	6.6	Right hemidiaphragm	Laparotomy	Mesh repair
48	Tong 2015 [55]	China	F	36	Lower back pain	17	Left upper retroperitoneal space	Laparotomy	
49	Trehan 2015 [56]	India	F	34	Heaviness in right flank	10	Right retroperitoneal hypochondrium	Laparoscopic	
50	Yoon 2015 [57]	South Korea	M	57	No	4.8	Left adrenal gland	Laparoscopic	
51	Pasquer 2016 [58]	France	M	36	No	2	Retro-rectal space	Sacrococcygeal	No recurrence after 3 years
52	Wang 2017 [13]	China	F	48	No	8	Left adrenal gland	Laparoscopic	
53	Byers 2018 [8]	USA	M	52	No	3.1	Left adrenal gland	FNA	Colon cancer
54	Liu 2018 [5]	China	M	33	No	4.5	Left hepatic hilum	Robotic surgery	CA19–9 312iu/m
55	Yuan 2021*	China	F	53	Back pain	3.5	Left adrenal gland	Laparoscopic	Normal follow-up

*NA* not available; *GEJ* gastroesophageal junction; *FNA* fine needle aspiration; *M* male; *F* female; *UK* United Kingdom; *USA* United States of American

*Our case

A similar literature review was conducted by Govaerts et al. in 2012, which concisely summarized 30 cases of true retroperitoneal bronchogenic cysts [2]. To our knowledge, our review provides the largest case series of adult patients with such cysts. Unfortunately, the exact pathogenesis of retroperitoneal bronchogenic cyst is still undetermined. More than 30 years ago, it was hypothesized that the pleuroperitoneal membrane and embryonic diaphragm might have not yet fused in an early development stage [1]. Consequently, abnormal tracheobronchial buds could be isolated by the growing diaphragm and migrate into abdominal cavity, which finally develop into bronchogenic cysts in the retroperitoneal space. Since the left pericardioperitoneal canal is larger and closes later than the right one, 80% of reported cases located in the left side of the retroperitoneal region, as in our reported case. According to the included cases (Table 1), the most common location of retroperitoneal bronchogenic cyst is near the left adrenal gland, and the second most common location is the peripancreatic region. There is a case of bilateral adrenal multilocular retroperitoneal bronchogenic cysts [9]. Retroperitoneal bronchogenic cyst occurs with equal frequency in men and women, with an average diagnosis age of 43.2 (range 17–69) years.

As mentioned above, the majority of reported retroperitoneal bronchogenic cysts were asymptomatic and discovered incidentally as our case. However, some specific cysts were discovered when causing certain symptoms, such as infection, uncomfortable abdomen and vomiting, from compression to adjacent organs. Patients usually complain epigastric pain or left flank pain when the cyst is over 5 cm in diameter. Up to date, Mirsadeghi et al. reported the largest retroperitoneal bronchogenic cyst in a 23-year-old man, with a maximal diameter of 20 cm [10].

In the current literature review, retroperitoneal bronchogenic cysts were confirmed after surgery or invasive biopsy. It remains quite difficult to make correct diagnosis preoperatively. Such cystic lesions would not arouse specific symptoms, therefore, they are easily misdiagnosed as non-neoplastic tumors, such as adrenal adenoma, adrenal cyst and pancreas cyst, or neoplastic tumors, such as adrenal cortical carcinoma, pheochromocytoma and pancreatic adenocarcinoma. Although CT scans and MRI examinations are helpful to locate the retroperitoneal masses, they play a minor role in determining the origin of such lesions. In clinical workup, a cystic mass in the retroperitoneal space could account for a broad diagnosis entity, including benign tumors such as lymphangioma, urothelial cyst, microcystic pancreatic adenoma, and other masses such as hematoma, abscess, duplication cyst, ovarian cyst and pancreatic pseudocyst, malignant tumors such as cystic mesothelioma, teratoma, undifferentiated sarcoma, cystic metastases (especially from ovarian or gastric adenocarcinomas), and malignant mesenchymoma [11].

To the present, histopathology is indispensable to clarify a definitive diagnosis of bronchogenic cyst. The essential pathological criteria are the presence of secretory respiratory lining epithelium along with seromucous glands, smooth muscle cells or hyaline cartilage [12]. Of note, all three important structures mentioned above were found in our reported case (Fig. 2).

The relationship between tumor markers and bronchogenic cysts is still unknown. Wang et al. reported a case of retroperitoneal bronchogenic cyst with serum CA 19-9 level more than 1200 U/mL. After 2 months of cyst excision, the CA 19-9 level returned to normal (cutoff: 35 U/mL) [13]. Another case with increased serum CA 19-9 level (144.1 U/mL) was reported by Choi et al. [14]. Besides, the CEA level was elevated at 3777 µg/L (cutoff: 5 µg/L) inside the cyst, but normal at 4.3 µg/L in the serum [6].

In order to confirm the diagnosis, relieve associated symptoms and prevent any potential malignant transformations, surgical resection is suggested for either symptomatic or asymptomatic retroperitoneal bronchogenic cyst [15]. Complete laparoscopic excision of cystic lesion is safe and feasible in our case. Moreover, it improves postoperative discomfort and shortens hospital stay as compared to traditional open approach. In recent years, robotic surgery, which could facilitate precise dissection of retroperitoneal tumors, was successfully applied to excise retroperitoneal bronchogenic cyst [5]. Generally, a complete resection via laparoscopic surgery often earns a good prognosis for retroperitoneal bronchogenic cysts, with no report of recurrence noted in this review.

In the current study, we reported a case with an ectopic bronchogenic cyst in the left retroperitoneal region. The uncommon ectopic cyst should be well known and considered as a part of differential diagnosis for a retroperitoneal neoplasm. A literature review suggests that retroperitoneal laparoscopic excision is an optimal management to establish both diagnosis and treatment. The long-term outcome of this disease is excellent, with no report of recurrence.

## Data Availability

The datasets used during the current study are available from the corresponding author on reasonable request.

## Abbreviations

CT: Computed tomography
MRI: Magnetic resonance imaging
CEA: Carcinoembryonic antigen
CA19-9: Carbohydrate antigen 19-9
CA125: Carbohydrate antigen 125
